# Voltage-Dependent Protonation of the Calcium Pocket Enable Activation of the Calcium-Activated Chloride Channel Anoctamin-1 (TMEM16A)

**DOI:** 10.1038/s41598-020-62860-9

**Published:** 2020-04-20

**Authors:** Guadalupe Segura-Covarrubias, Iván A. Aréchiga-Figueroa, José J. De Jesús-Pérez, Alfredo Sánchez-Solano, Patricia Pérez-Cornejo, Jorge Arreola

**Affiliations:** 1 Division de Biología Molecular del Instituto Potosino de Investigación Científica y Tecnológica. Camino a la Presa de San José 2055, San Luis Potosí, SLP 78216 México; 2 Physics Institute, Universidad Autónoma de San Luis Potosí, Ave. Dr. Manuel Nava #6, San Luis Potosí, SLP 78290 México; 3 Department of Physiology and Biophysics, Universidad Autónoma de San Luis Potosí School of Medicine, Ave. V. Carranza 2405, San Luis Potosí, SLP 78290 México

**Keywords:** Biophysics, Physiology

## Abstract

Anoctamin-1 (ANO1 or TMEM16A) is a homo-dimeric Ca^2+^-activated Cl^−^ channel responsible for essential physiological processes. Each monomer harbours a pore and a Ca^2+^-binding pocket; the voltage-dependent binding of two intracellular Ca^2+^ ions to the pocket gates the pore. However, in the absence of intracellular Ca^2+^ voltage activates TMEM16A by an unknown mechanism. Here we show voltage-activated anion currents that are outwardly rectifying, time-independent with fast or absent tail currents that are inhibited by tannic and anthracene-9-carboxylic acids. Since intracellular protons compete with Ca^2+^ for binding sites in the pocket, we hypothesized that voltage-dependent titration of these sites would induce gating. Indeed intracellular acidification enabled activation of TMEM16A by voltage-dependent protonation, which enhanced the open probability of the channel. Mutating Glu/Asp residues in the Ca^2+^-binding pocket to glutamine (to resemble a permanent protonated Glu) yielded channels that were easier to activate at physiological pH. Notably, the response of these mutants to intracellular acidification was diminished and became voltage-independent. Thus, voltage-dependent protonation of glutamate/aspartate residues (Glu/Asp) located in the Ca^2+^-binding pocket underlines TMEM16A activation in the absence of intracellular Ca^2+^.

## Introduction

Anoctamin-1 (ANO1 or TMEM16A) and Anoctamin-2 (ANO2 or TMEM16B) are the pore-forming subunits of Ca^2+^-activated Cl^−^ channels (CaCCs)^1–3^. Several tissues express CaCCs that participate in vital physiological functions^4,5^. Thus, a role for TMEM16A and TMEM16B in smooth muscle contraction, control of blood pressure, control of gastrointestinal movements, regulation of cardiac and neuronal excitability, fluid secretion in exocrine glands, secretion of melatonin, mucin and insulin, sperm capacitation and motility, inhibition of polyspermy, and sensory transduction was established using tissue-specific knockout mice^6–16^. In addition, TMEM16A modulates the partitioning of membrane phosphoinositides and endocytic transport by controlling the [Cl^−^]_i_^17^. Overexpression of TMEM16A is associated with hypertension, increased cell proliferation and cancer progression^18–21^.

Activation of CaCCs is triggered by voltage-dependent binding of two Ca^2+^ ions to the channel when the intracellular Ca^2+^ concentration ([Ca^2+^]_i_) increases^22–26^. Structural and mutagenesis analysis show that Ca^2+^ ions bind to an acidic Ca^2+^ pocket formed by four Glu, one Asp and one Asn^25–27^. The pocket is located near the cytosolic side facing the permeation pathway. However, other divalent cations and maybe trivalent cations too can support TMEM16A activation. Based on the cation concentrations to obtain the half-maximum response, the cation selectivity of TMEM16A gating machinery is Ca^2+^»Sr^2+^»Ba^2+^»Cd^2+^,^23,27–29^. Gd^3+^ may also activate TMEM16A since its application removed the inward rectification of the Gly644Pro TMEM16A mutant channel^30^. Mg^2+^, the most abundant divalent cation in the cytoplasm of our cells^31^ is unable to activate TMEM16A, however, Mg^2+^ competes with Ca^2+^ and decreases the apparent Ca^2+^ sensitivity of TMEM16A^29^. Despite its low cation selectivity, the Ca^2+^-binding pocket of TMEM16A does not interact with monovalent cations. Due to its acidic chemical nature, the Ca^2+^-binding pocket is prompt to protonation. In fact, intracellular H^+^ compete with Ca^2+^ for these acidic residues. By doing so, H^+^ decreases the Ca^2+^ affinity of TMEM16A causing a reduction in channel activity^32,33^. Thus, intracellular H^+^ interact with the pocket, but can this interaction prompt TMEM16A activation? In this work, we demonstrate that voltage activation of TMEM16A in the absence of intracellular Ca^2+^ is due to voltage-dependent titration of the Ca^2+^-binding pocket. In the absence of Ca^2+^ voltage induced strong outwardly rectifying anion currents sensitive to TMEM16A inhibitors. These currents activate and deactivate very fast (<1 ms) and displayed little time dependence. Furthermore, we show that Ca^2+^-independent activation of TMEM16A under acidic conditions resulted from voltage-dependent protonation of Glu/Asp residues.

## Results

### Voltage activation of TMEM16A in the absence of intracellular calcium

**Figure 1:**
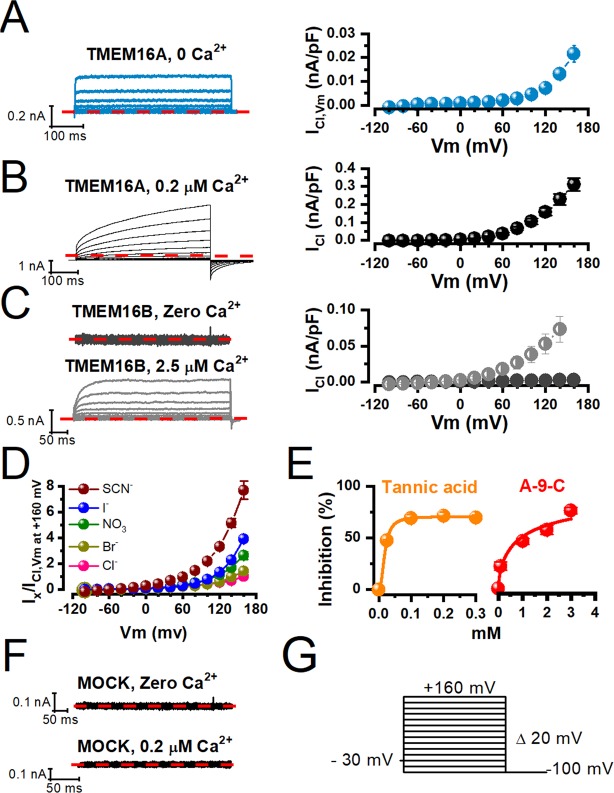
Activation of TMEM16A by voltage in the absence of intracellular calcium. (**A**) Left: Representative I_Cl,Vm_ recordings obtained from a HEK-293 cell expressing WT TMEM16A and dialysed with 25.24 mM EGTA/0 Ca^2+^. Right: I_Cl,Vm_ - Vm relationship for WT TMEM16A I_Cl,Vm_ density (n = 10). [Cl^−^Cl^−^]_e_/[Cl^−^]_i_ = 140/40 mM, pH_e_/pH_i_ = 7.3. (**B**) Representative I_Cl_ recording obtained from a HEK-293 cell expressing WT TMEM16A and dialysed with 0.2 µM Ca^2+^. Right: I_Cl_ - Vm relationship constructed with WT TMEM16A I_Cl_ density (n = 10). [Cl^−^]_e_/[Cl^−^]_i_ = 140/40 mM, pH_e_/pH_i_ = 7.3. (**C**) Representative I_Cl_ recordings obtained from two different HEK-293 cells expressing TMEM16B that were dialysed with 0 Ca^2+^ + 25.24 mM EGTA (upper left) or 2.5 µM Ca^2+^ (lower, left). Right: I_Cl_ - Vm relationships constructed with the TMEM16B current density in the absence (black) and in the presence (grey) of 2.5 µM Ca^2+^. [Cl^−^]_e_/[Cl^−^]_i_ = 140/40 mM, pH_e_/pH_i_ = 7.3 (n = 5). (**D**)Anion selectivity of WT TMEM16A I_Cl,Vm_ when [Ca^2+^]_i_ = 0. The extracellular Cl^−^ (140 mM) was replaced by the indicated anions. Paired I_Cl,Vm_ − Vm relationships in the presence of Cl^−^ (control) and then in the presence of a chosen anion (test) were obtained from the same cell. Both relationships were normalized using I_Cl,Vm_ recorded in the presence of Cl^−^ at +160 mV and averaged. (n = 5–8). (**E**) Concentration-response curves to tannic and anthracene-9-carboxylic acids at +160 mV. Cells were dialyzed with 25.24 mM EGTA/0Ca^2+^ and bathed in 140 mM SCN^−^ media to increase I_Cl,Vm_ size. Continuous lines are fits to Hill´s equation with IC_50_/N/R^2^ values of 18.2 ± 1.7 µM/2.2/0.999 (n = 3–4) and 0.98 ± 0.22 mM/0.65/0.967 (n = 4), respectively. (**F**) Representative recordings obtained from HEK-293 cells transfected with the empty pIRES-II-EGFP vector in the absence (upper left) and in the presence of 0.2 µM Ca^2+^ (lower left). [Cl^−^]_e_/[Cl^−^]_i_ = 140/40 mM, pH_e_/pH_i_ = 7.3, (n = 5). (**G**) The voltage protocol used to activate TMEM16A consisted of a holding potential of −30 mV, 250 or 500 ms steps between −100 to +160 mV in 20 mV increments, and unless otherwise indicated, a repolarization Vm of −100 mV.

To study voltage-dependent gating of TMEM16A in the absence of intracellular Ca^2+^, we recorded whole cell currents from HEK-293 cells expressing TMEM16A dialyzed with a solution containing 25.24 mM EGTA without Ca^2+^. Under this condition, a current induced by voltage (Vm) is likely to result from gating of TMEM16A and we will refer to it as Vm-activated TMEM16A current or I_Cl,Vm_. Figure 1A shows a set of I_Cl,Vm_ recorded from a cell stimulated with the Vm protocol shown in Fig. 1G. At positive Vm, I_Cl,Vm_ activates and deactivates very fast. The time constant of activation was 0.59 ± 0.11 ms at +160 mV (n = 5); no tail currents were recorded upon repolarizing at −100 mV. The magnitude of I_Cl,Vm_ was constant during the entire stimulus duration. This behaviour contrasted with the classical time-dependent I_Cl_ generated upon depolarization and the corresponding large tail currents induced by repolarizing to −100 mV in the presence of 0.2 µM Ca^2+^ (Fig. 1B). The corresponding I_Cl,Vm_ - Vm curves show that TMEM16A displays strong outward rectification in the absence of intracellular Ca^2+^ (blue). The magnitude of I_Cl,Vm_ was small; at +160 mV, it was about 10 times smaller than that observed in the presence of 0.2 µM Ca^2+^ (blue vs black). In contrast, TMEM16B, a paralog of TMEM16A, was activated in cells dialysed with 2.5 µM Ca^2+^ but not in cells dialysed with 25.24 mM EGTA and 0 Ca^2+^ (Fig. 1C). To corroborate that TMEM16A is anion-selective in 0 Ca^2+^ we determined the anion-dependence of I_Cl,Vm_. Figure 1D displays I_Cl,Vm_ - Vm relationships (left) obtained from cells dialysed with 0 Ca^2+^ and exposed to the indicated anions on the extracellular side. Because I_Cl,Vm_ displays strong outward rectification, we could not reliably measure reversal potentials to determine the anion selectivity sequence from permeability ratios. However, by taking the ratio of I_X,Vm_ (the current carried by anion X) relative to I_Cl,Vm_ at +160 mV we obtained the following anion selectivity sequence: SCN^−^ (7.7 ± 0.70; n = 6), I^−^ (3.9 ± 0.16; n = 5), NO_3_^−^ (2.6 ± 0.29; n = 5), Br^−^(1.38 ± 0.22; n = 5), and Cl^−^ (1.0; n = 10). This sequence was the same as that obtained in the presence of 0.2 µM Ca^2+^ (Supplementary Fig. S1). Next, we examined the sensitivity of I_Cl,Vm_ to tannic and anthracene-9-carboxylic (A-9-C) acids, blockers of TMEM16A. Figure 1E shows concentration-response curves at +160 mV obtained from cells dialysed with 0 Ca^2+^ solutions and exposed to increasing concentrations of tannic and A-9-C acids. I_Cl,Vm_ was inhibited with IC_50_ values of 18.2 ± 1.7 µM and 0.98 ± 0.22 mM, respectively. The IC_50_ values determined for both inhibitors are like those reported in the presence of Ca^2+^ ^34,35^. Finally, we recorded from mock-transfected HEK-293 cells dialyzed with 0 or 0.2 µM Ca^2+^ but we did not observe endogenous currents activated by Vm (Fig. 1F). Taken together, these results show that Vm activates TMEM16A in the absence of intracellular Ca^2+^.

### Voltage-dependent protonation of TMEM16A enables activation

**Figure 2:**
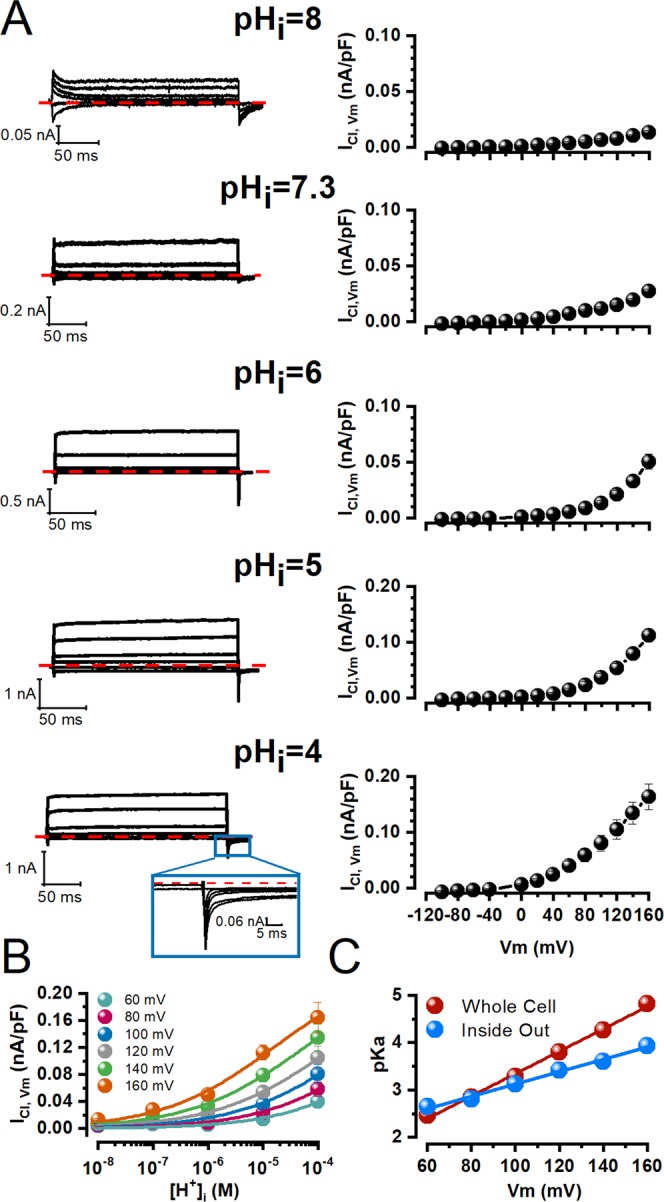
Voltage-dependent protonation facilitates activation of TMEM16A in the absence of intracellular calcium. (**A**) Representative I_Cl,Vm_ recordings (left) and corresponding I_Cl,Vm_ - Vm relations (right) sampled from different cells dialyzed with an internal solution whose pH was set to (from top to bottom): 8.0 (n = 4), 7.3 (n = 8), 6.0 (n = 6), 5.0 (n = 5), and 4.0 (n = 5). Current traces were recorded at −100, −40, +20, +60, +120 and +160 mV. An amplification of the tail currents at pH_i_ 4.0 is shown. [Cl^−^]_e_/[Cl^−^]_i_ were 140/40 mM and pH_e_ = 7.3 for all cases. (**B**) Titration curves at the indicated Vm. Continuous lines are fits with the Hill equation (Eq. 1) using a single maximum current density value of 0.23853 nA/pF determined by fitting the curve at +160 mV. The equilibrium constant of titration *K* and the Hill coefficient N were determined from the fits. The *K/N/R*^2^ values were 0.0035 ± 0.002 M/0.44 ± 0.07/0.951 at 60 mV, 0.0014 ± 0.00075 M/0.43 ± 0.06/0.96 at +80 mV, 0.00052 ± 0.00016 M/0.42 ± 0.04/0.981 at +100 mV, 0.000159 ± 0.000029 M/0.42 ± 0.03/0.990 at +120 mV, 0.000055 ± 0.00000071 M/0.42 ± 0.02/0.994 at +140, and 0.0000152 ± 0.0000016 M/0.43 ± 0.076/0.990 at +160 mV. (**C**) Vm dependence of the equilibrium constant of protonation *K* determined from data shown in B. Continuous line is the fit with Eq. 2 with a δ value of 1.39 ± 0.04 and pK_0_ of 0.97 ± 0.07. For comparison, we plotted in blue average *pKa* values calculated from two titration curves obtained from two inside out patches. The continuous blue line is the fit with Eq. 2 with a δ value of 0.77 ± 0.03 and pK_0_ of 1.82 ± 0.06.

Under physiological [Ca^2+^]_i_ and [H^+^]_i_, TMEM16A is gated by Vm-dependent binding of intracellular Ca^2+^ to the Ca^2+^-binding pocket^22–25^. However, intracellular acidification in the presence of Ca^2+^ inhibited Ca^2+^-activated Cl^−^ currents in salivary acinar cells and HEK-293 cells expressing TMEM16A by competing for high-affinity binding sites in the Ca^2+^-binding pocket^32,33^. Based on this observation we hypothesized that positive Vm could drive intracellular H^+^ into the Ca^2+^-binding pocket to protonate Glu/Asp residues, open the channel and thus generate I_Cl,Vm_. To test this idea, we recorded I_Cl,Vm_ from TMEM16A-expressing HEK-293 cells dialyzed with an internal solution containing 25.24 mM EGTA/0 Ca^2+^. The pH of this solution was adjusted to 8.0, 7.3, 6.0, 5.0, and 4.0 thus changing the [H^+^]_i_ by four-orders of magnitude. To ensure that pH_i_ remained constant during our recordings, we increased the buffer capacity of the solutions by adjusting the pH with 50 mM of bicine (8.0), HEPES (7.3), MES (6.0, 5.0, and 4.0), citric acid (5.0), or tartaric acid (4.0). No difference in channel activation was observed between data obtained with different buffers at the same pH. Figure 2A shows I_Cl,Vm_ recorded at −100, −40, +20, +60, +120 and +160 mV using the protocol shown in Fig. 1G. Each row shows I_Cl,Vm_ recorded at the indicated pH (left column), traces are representative of 5 independent experiments. As pH_i_ decreased from 8.0 (top) to 4.0 (bottom), the magnitude of I_Cl,Vm_ increased. Under acidic conditions I_Cl,Vm_ activated and deactivated very rapidly. At +160 mV, the time constant of activation was 0.24 ± 0.02 ms at pH_i_ = 4 (n = 5). Tail currents were nearly absent but at pH_i_ 4.0 a small and fast inward current became evident (inset shows a magnification of the tail current). The corresponding I_Cl,Vm_ - Vm relationships are shown on the right column of Fig. 2A. At −100 mV a reduction of pH_i_ from 6 to 4.0 produced a 5-fold increase in I_Cl,Vm_ (−1.5 ± 0.4 to −7.5 ± 2.7 pA/pF, n = 5. Lastly, no currents were recorded from HEK-293 cells transfected with the empty vector or dialyzed with pH_i_ 4.0 containing EGTA or BAPTA (Supplementary Fig. S2). Hence, the potentiation of I_Cl,Vm_ under acidic conditions resulted from activation of TMEM16A. Unfortunately, the effect of intracellular H^+^ on the Vm-dependent activation of I_Cl,Vm_ cannot be deduced from the analysis of macroscopic conductance (G = I_Cl,Vm_/Vm-Vr) vs Vm at different [H^+^]_i_ for two reasons. First, the reversal potentials (Vr) measurements at pH_i_ ≥ 6.0 were unreliable due to I_Cl,Vm_ rectification (at pH_i_ 5 and 4, reversal potentials were −34.4 ± 2.3 and −31.4 ± 0.8 mV, respectively, closer to the expected reversal potential for Cl^−^). Second, the instantaneous current-voltage relationship after a depolarization to +160 mV was not linear (Supplementary Fig. S3, blue) indicating that I_Cl,Vm_ does not follow Ohm´s law and the tail current magnitudes at −100 mV (orange) were the same for steps between −100 to +160 mV as if the open probability was Vm-independent.

To advance the hypothesis that Vm activation of TMEM16A is due to protonation we must show that the equilibrium constant of protonation (*K*) is Vm-dependent. Figure 2B show I_Cl,Vm_ titration curves at different Vm. As the [H^+^]_i_ increase the magnitude of I_Cl,Vm_ is increased. Unfortunately, only at +160 mV and pH_i_ 4.0, we obtained a hint of saturation. Experiments at pH_i_ 3.0 were not possible because the cells died quickly. Thus, to determine the equilibrium constant of protonation (*K*) at each Vm, we first fitted the data collected at +160 mV to a Hill equation (Eq. 1) to obtain an I_Cl,Vm_ maximum. Then we used this value in the Hill equation to fit all the curves. This way, our fitting procedure had only two free parameters, *K* and Hill coefficient (N). Continuous lines in Fig. 2B and their corresponding R^2^ values show that this fitting procedure is a good quantitative description of the experimental data at all Vm. Figure 2C displays the resulting *pKa* (= −log_10_ *K*) values as a function of Vm. At positive Vm, the *pKa* value increased indicating that less H^+^ are required to generate 50% of I_Cl,Vm_. This Vm dependence of *pKa* resembles the Vm dependence of EC_50_ for intracellular Ca^2+^ ^22^. The relation *pKa* – Vm was fit with Eq. 2 to calculate *K*_0_ (equilibrium constant of protonation at 0 mV and δ, the fraction of electrical field sensed by H^+^ going from the intracellular side to the extracellular side). A *pK*_0_ value of 0.97 ± 0.07 and a δ value of 1.39 ± 0.04 were estimated this way. We conducted similar experiments with inside-out patches; unfortunately, most patches were unstable at acidic conditions. Figure 2C (blue symbols) shows averaged data obtained from two super-patches (Supplementary Fig. S4) that withstood exposure to pH_i_ 8.0, 6.0, 5.0, and 4.0. The *pKa* obtained from inside-out patches has a similar Vm-dependence as the *pKa* obtained from whole cell recordings. In this case, the average *pK*_0_ and δ values were 1.77 and 0.79, respectively. Thus, taken together our results support the hypothesis that Vm gates TMEM16A due to intracellular protonation.

### Voltage-dependent protonation of glutamate and aspartate residues located in the Ca^2+^-binding pocket increase the open probability of TMEM16A

**Figure 3:**
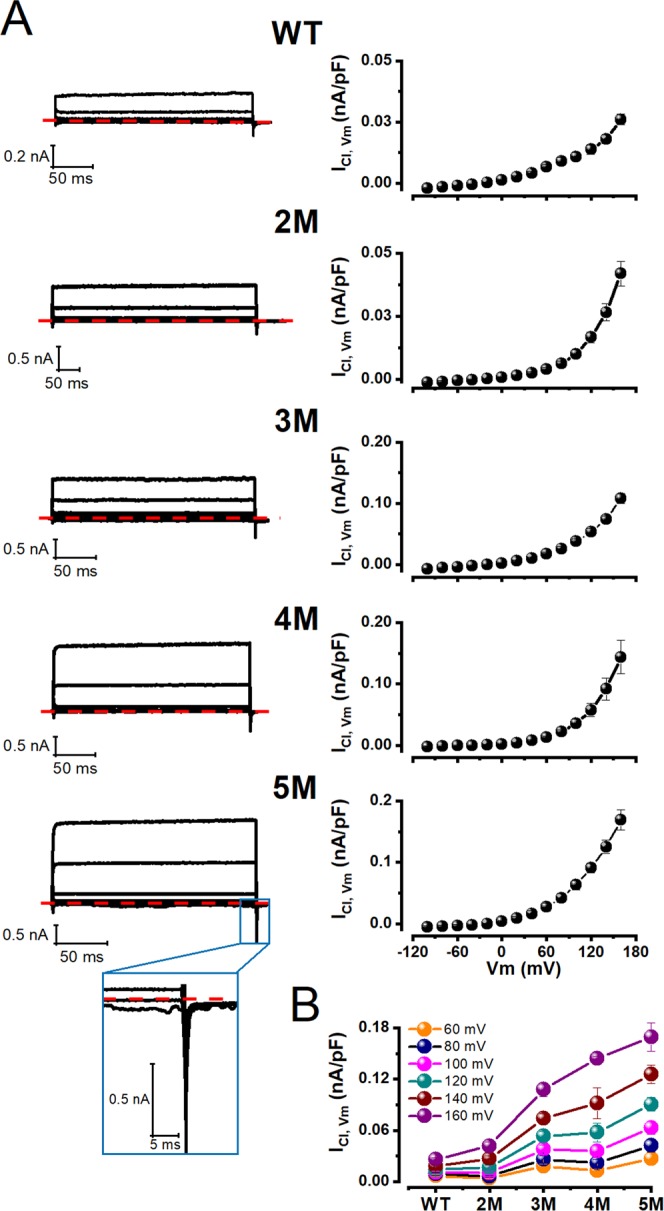
Mutating acidic residues (Glu to Gln) from the Ca^2+^-binding pocket enhanced the voltage activation of TMEM16A at physiological intracellular pH. (**A**) Representative I_Cl,Vm_ recordings (left) sampled from different cells expressing from top to bottom WT (n = 8), 2 M (n = 5), 3 M (n = 6), 4 M (n = 6), and 5 M (n = 6) TMEM16A channels and their corresponding I_Cl,Vm_ - Vm relations (right). Note the absence of tail currents in these recordings (Inset: tail currents from 5 M). In all recordings: [Cl^−^]_e_/[Cl^−^]_i_ = 140/40 mM, pH_e_/pH_i_ = 7.3/7.3 and [Ca^2+^]_i_ = 0. (**B**) Current density vs the number of acidic residues mutated into Gln at different Vm.

To identify Glu and Asp residues within the Ca^2+^-binding pocket that are targets of intracellular H^+^ we mutated in an incremental fashion 4 Glu and 1 Asp residues. These residues were mutated into Gln to resemble a permanent protonated Glu side chain. We reasoned that if H^+^ neutralizes the COO^−^ group of Glu and Asp residues to activate wild type TMEM16A (WT) then Vm would activate Gln mutants at pH_i_ 7.3 without the need for acidification. Figure 3A shows I_Cl,Vm_ recordings at −100, −40, +20, +60, +120 and +160 mV from five independent cells expressing (from top to bottom) WT, E702Q/E705Q (2 M), E702Q/E705Q/E734Q (3 M), E702Q/E705Q/E734Q/D738Q (4 M), and E654Q/E702Q/E705Q/E734Q/D738Q (5 M) channels. All cells were dialyzed with 25.24 mM EGTA/0 Ca^2+^ and pH_i_ 7.3. As the number of mutations accumulates, the magnitude of I_Cl,Vm_ increased mirroring the effect of intracellular acidification. I_Cl,Vm_ onset was fast, had no time dependence and no tail currents at −100 mV. Very fast tail currents were observed only with the quintuple mutant (see magnification in the lower-left panel). The corresponding I_Cl,Vm_ - Vm relationships (Fig. 3A, right column) displayed outward rectification just like WT channels but the rectification changed without showing a particular pattern. Although the magnitude of I_Cl,Vm_ increased at all Vm, at +160 mV I_Cl,Vm_ appeared to saturate as the number of mutations (or Gln) in the Ca^2+^-binding pocket increased (Fig. 3B). At pH_i_ 7.3, a strong depolarization (+160 mV) induced a 6.5-fold increase in I_Cl,Vm_ in 5 M channels compared to WT.

**Figure 4:**
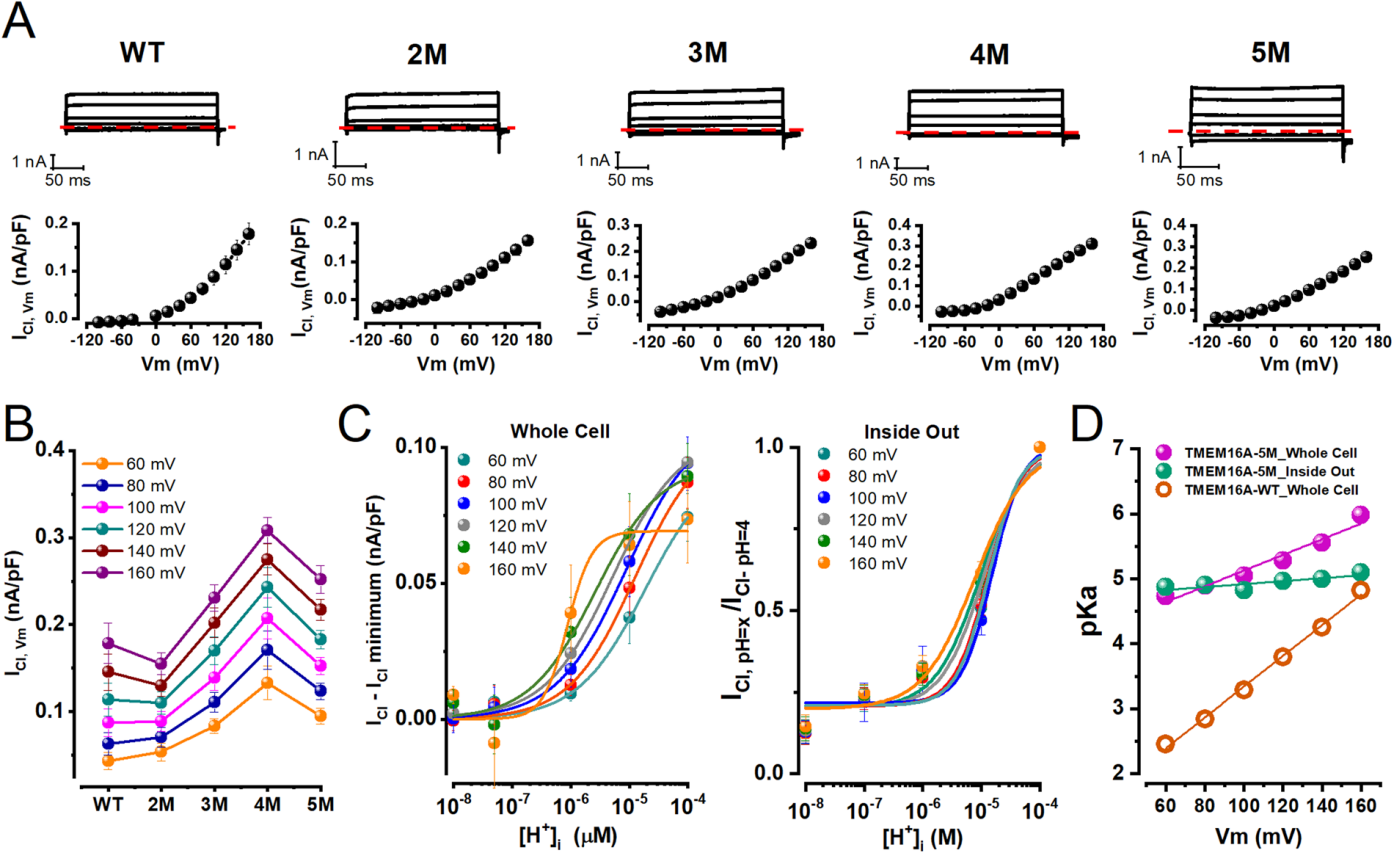
Ca^2+^-binding pocket mutants are less sensitive to intracellular acidification. (**A**) Representative I_Cl,Vm_ recordings (upper panels) and their corresponding I_Cl,Vm_ - Vm relations (lower panels). From left to right: WT (n = 5), 2 M (n = 5), 3 M (n = 5), 4 M (n = 6), and 5 M (n = 6) TMEM16A channels. I_Cl,Vm_ were sampled from different cells dialyzed with an internal solution whose pH was set to 4.0 and [Ca^2+^]_i_ = 0. Note the presence of tiny tail currents. [Cl^−^]_e_/[Cl^−^]_i_ = 140/40 mM, pH_e_/pH_i_ = 7.3/4.0 and [Ca^2+^]_i_ = 0 in all cases. (**B**) Current density vs the number of acidic residues mutated into Gln at different Vm. (**C**) Titration curves of 5 M TMEM16A channels in the range of +60 to +160 mV. Curves were constructed using whole cell I_Cl,Vm_ (left) or inside-out I_Cl,Vm_ (right) data and then fitting the curves with the Hill equation (Eq. 1, continuous lines) to determine *K* values at each Vm. (**D**) The Vm dependence of *pKa* was dampened in 5 M TMEM16A channels. Purple and green spheres show *pKa* values at different Vm calculated using the whole cell and inside-out data, respectively. Continuous lines are fits with Eq. 2 to obtain *pK*_*O*_ and the δ, the fraction of electrical distance. The corresponding values for whole cell and inside out data were 3.91 ± 0.14/0.71 ± 0.72 and 4.69 ± 0.08/0.13 ± 0.04, respectively. For comparison, the Vm dependence of WT *pKa* is shown in orange.

The above data show that mutating the acidic residues of the Ca^2+^-binding pocket into Gln allowed activation of TMEM16A at pH_i_ 7.3 in the absence of intracellular Ca^2+^. If these residues are the main source of Vm sensitivity under acidic conditions, then increasing the [H^+^]_i_ should have little or no additional effect on I_Cl,Vm_, and the Vm dependence of *pKa* should be abolished. To test these predictions, we recorded I_Cl,Vm_ from cells dialyzed with an intracellular solution with pH_i_ 4. Figure 4A shows I_Cl,Vm_ recordings for WT, 2 M, 3 M, 4 M, and 5 M channels (left to right). Current kinetics of I_Cl,Vm_ at pH_i_ 4.0 and 7.3 (compare Fig. 4A to Fig. 3A) were quite similar. For example, at +160 mV the time constant of activation of 5 M channels was 0.46 ± 0.04 ms (n = 5) at pH_i_ 7.3 and 0.77 ± 0.03 ms (n = 5) at pH_i_ 4.0. The corresponding I_Cl,Vm_ - Vm curves displayed less rectification (Fig. 4A, bottom panels). Reversal potential values for WT, 2 M, 3 M, 4 M, and 5 M, were −31.4 ± 0.8, −31.5 ± 0.7, −29.2 ± 0.7, −31.0 ± 2.1, and −28.0 ± 0.9 mV, respectively, indicating that at pH_i_ 4.0 the I_Cl,Vm_ was generated by Cl^−^ fluxes. However, at pH_i_ 4.0 the magnitude of I_Cl,Vm_ recorded at positive Vm increased with the number of mutations present (Fig. 4B). A caveat of using pH_i_ 4.0 to test protonation of Glu and Asp residues is that the probability of protonation is about 0.64, therefore, comparing WT and mutant channels at pH_i_ 4.0 may not be straightforward. Despite this limitation and contrary to the result obtained at pH_i_ 7.3 (Fig. 3B), Vm activation of I_Cl,Vm_ at pH_i_ 4.0 was strongly weakened but not eliminated. Depolarizing to +160 mV induced a 1.4-fold increase in I_Cl,Vm_ generated by 5 M channels compared to WT at pH_i_ 4.0. Thus, mutating 4 Glu and 1 Asp located in the Ca^2+^-binding pocket of TMEM16A reduced but did not completely abolish the ability of intracellular H^+^ to increase I_Cl,Vm_.

To investigate if the Vm dependence of *pKa* was eliminated by mutating the acidic residues we performed concentration-response experiments in 5 M channels. Figure 4C shows titration curves constructed using the I_Cl,Vm_ recorded between +60 to +160 mV from the whole cell (left) or inside-out patches (right). Unlike WT channels (Fig. 2B), the 5 M mutant channels display nearly overlapping titration curves at different Vm indicating less Vm dependence. This behaviour was more evident for inside-out patch data possibly because we were able to test all [H^+^]_i_s in each patch. Curves were fit with the Hill equation to calculate *pKa*. Figure 4D (purple = whole cell; green = inside-out patches) shows *pKa* values against Vm. *pKa* values fall in a range comprising 4.7 to 6 (note a smaller range for inside-out patch data), indicating that residues other than Glu and Asp are being protonated. These *pKa* – Vm curves were fitted with Eq. 2 to estimate *pK*_0_ and Vm sensitivity. *pK*_0_*/δ* values were 4.69 ± 0.08/0.13 ± 0.04 (inside out) and 3.91 ± 0.14/0.70 ± 0.07 (whole cell). Thus, compared to WT (replotted in orange) protonation in 5 M channels is less Vm-dependent. This loss of Vm dependence is also illustrated by the fact that this channel is partially open at a low [H^+^]_i_. Thus, mutating the acidic residues decreased the Vm dependence of TMEM16A titration lending support to the idea that the Ca^2+^-binding pocket is the target of intracellular H^+^. In addition, the activity of TMEM16A-5M channels was enhanced under acidic conditions by a weakly Vm-dependent protonation mechanism.

**Figure 5:**
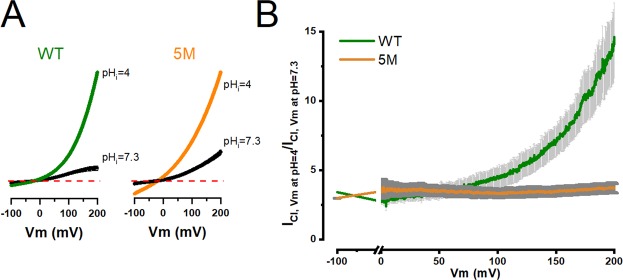
Intracellular acidification enables activation of TMEM16A by increasing the apparent open probability in Vm-dependent and Vm-independent manners. (**A**) I_Cl,Vm_ - Vm relationships recorded from inside-out patches obtained from HEK-293 cells expressing TMEM16A WT (left panel) or TMEM16A 5 M (right panel). The cytosolic side of each patch was sequentially exposed to solutions pH 7.3 and then 4.0. I_Cl,Vm_ - Vm curves were generated applying a ramp protocol that varied Vm between −100 and 200 mV in 624 ms. Paired curves were normalized to the current obtained at +200 mV and pH_i_ = 4.0. Normalized relations were then averaged. The reversal potentials were: −36.2 ± 1.4 at pH_i_ = 7.3 and −31.3 ± 1.4 at pH_i_ = 4.0 for WT and −28.3 ± 1.5 at pH_i_ = 7.3 and −23.4 ± 1.6 at pH_i_ = 4 for 5 M. [Cl^−^]_e_/[Cl^−^]_i_ = 140/40 mM, pH_e_ = 7.3 and [Ca^2+^]_i_ = 0 in all cases. (**B**) Ratio of I_Cl,Vm_ recorded at pH_i_ 4.0 to I_Cl,Vm_ recorded at pH_i_ 7.3 utilized to determine the open probability at each Vm after intracellular acidification. Enhancement of open probability for WT channels (green line; n = 10) and 5 M channels (orange line; n = 13) was induced by acidification to pH_i_ = 4.0.

To further understand the mechanism by which intracellular H^+^ activates TMEM16A, we investigated whether protonation of the Ca^2+^-binding pocket increases the open probability. To this end, we recorded I_Cl,Vm_ - Vm curves from inside-out patches consecutively exposed to pH_i_ 7.3 and 4.0. We used a ramp protocol that changed the Vm between −100 to +200 mV in 635 ms. pH_i_ 4.0 was chosen because under this acidic condition 64% of the time the Glu side chain will be in the protonated state, assuming the *pKa* value of Glu is 4.25. Figure 5A shows two sets of I_Cl,Vm_ - Vm curves recorded from patches excised from a cell expressing WT (left) or 5 M (right) channels. At pH_i_ 7.3, the WT channels displayed a small I_Cl,Vm_ at all Vm range. However, upon exposure to a solution pH_i_ 4.0 the I_Cl,Vm_ was strongly enhanced resulting in an outward rectifying current. Similarly, in 5 M channels (right) I_Cl,Vm_ increased further when the pH_i_ went from 7.3 to 4.0 with little alteration in the reversal potentials (ΔVr= 4.95 ± 0.8 for WT and ΔVr = 4.9 ± 0.9 mV for 5 M). To determine whether the open probability was enhanced by acidification, we calculated the ratio of I_Cl,Vm_ at pH_i_ 4.0 and 7.3 (I_pHi = 4_/I_pHi = 7.3_). This value is directly proportional to the changes in the open probability induced by the acidification at each Vm. These ratios are shown in Fig. 5B. In WT channels the open probability (green) increased 3-fold at 0 mV and 14-fold at 200 mV, thus highlighting the effect of Vm-dependent protonation on open probability. However, the ratio I_pHi = 4_/I_pHi = 7.3_ for 5 M channels was Vm-independent (orange), albeit acidification produced a 3.5-fold increase in the open probability in the −100 to +200 mV range. Therefore, protonation is enough to grant Vm-dependent activation to TMEM16A channels in the absence of intracellular Ca^2+^. Also, the titration data indicates that TMEM16A activity is enhanced by Vm-independent protonation.

**Figure 6:**
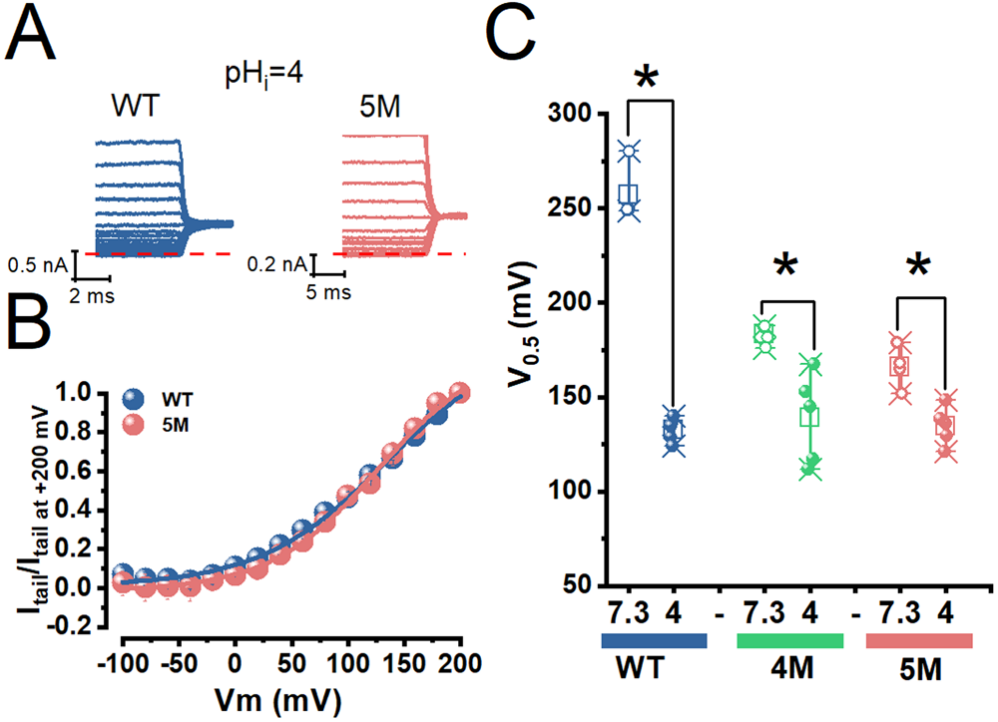
The acidic residues of the Ca^2+^-binding pocket control the voltage activation of TMEM16A. (**A**) Representative tail currents recorded at +100 mV from two cells expressing WT (blue) and 5 M (pink) channels. For clarity, we only plot the last 10 ms of the currents generated by depolarizing pulses. Currents were recorded using a P/8 protocol from cells dialyzed with an internal solution with pH = 4.0. [Cl^−^]_e_/[Cl^−^]_i_ = 140/40 mM, pH_e_ = 7.3 and [Ca^2+^]_i_ = 0 in all cases. (**B**) Corresponding activation curves constructed with the tail currents shown in A. Initial tail current magnitudes were measured and normalized to the value obtained at +200 mV. Continuous lines are fits with the Boltzmann Equation. V_0.5_ = 134.14 ± 9.8 mV and z = 0.02 for WT and V_0.5_ = 126.6 ± 5.6 mV and z = 0.02 for 5 M. (**C**) V_0.5_ values for WT (blue, n = 4), 4 M (green, n = 4), and 5 M (pink, n = 4) channels determined at pH_i_ 7.3 (open symbols) and 4.0 (closed symbols n = 5 all cases). V_0.5_ values were determined as shown in A and B. V_0.5_ values for WT at pH_i_ 7.3 (n = 4) were estimated by fitting the I_Cl,Vm_ - Vm curves with Eq. 3. Statistical differences were showed with an asterisk, p = 0.04.

The data described show that the activation of TMEM16A in the absence of intracellular Ca^2+^ is enhanced by mutating the acidic residues of the Ca^2+^-binding pocket. We took advantage of the strong activation of 4 M and 5 M channels at pH_i_ 7.3 to record tail currents to analyse the effect of acidification on voltage-dependent activation. We recorded tail currents at +100 mV using a P/8 protocol in cells expressing WT, 4 M and 5 M channels dialyzed with an internal solution pH_i_ = 4.0. Because I_Cl,Vm_ reaches steady-state very fast, we employed 20 ms depolarization pulses (Fig. 6A). The Vm-dependent activation was measured by the V_0.5_ value (determined from Boltzmann equation fits to the normalized tail current vs Vm curves). V_0.5_ values were the same for WT, 5 M and 4 M channels (Fig. 6A–C); these values were in the range 129–140 mV (WT), 111–167 mV (4 M), and 117–149 mV (5 M). At pH_i_ 7.3, V_0.5_ values were more positive for the three channels, indicating that acidification shifted the Vm dependence of activation. Although the V_0.5_ values for 4 M and 5 M channels were on average 17 mV apart at pH_i_ 7.3, the statistical analysis indicated that at p < 0.04 the values were not different. However, this tendency and the effect of pH_i_ 4.0 on 4 M channels suggest that protonating or mutating residue E654 facilitates voltage activation. The estimated V_0.5_ value for activation of WT channels at pH_i_ 7.3 was obtained by fitting the current-voltage relations with Eq. 3. The resulting values are plotted as open blue symbols in Fig. 6C. By comparing this result with that obtained at pH_i_ 4.0 (closed blue symbols) we can conclude that intracellular protons shifted the Vm activation by more than −100 mV.

## Discussion

**Figure 7:**
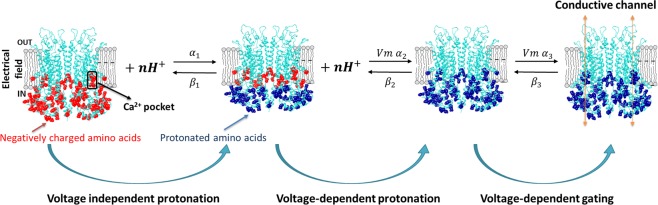
Schematic representation of the proposed mechanism of TMEM16A activation by voltage-independent and voltage-dependent protonation. We advocate that voltage activation of TMEM16A in the absence of intracellular Ca^2+^ by protonation proceeds in three steps. The first and second are voltage-independent and voltage-dependent protonation steps, respectively. Channel opening is achieved by a voltage-dependent transition occurring during the last step. α_1_, α_2_, and α_3_ are forward rate constants whereas β_1_, β_2_ and β_3_ are backward rate constants. TMEM16A is depicted in blue embedded in a grey membrane. Red dots are intracellular un-protonated acidic residues that are potential targets of intracellular protons. Residues outside the electrical field are protonated in a voltage-independent manner (navy). Inside the black rectangle are the acidic residues of the Ca^2+^ pocket (one subunit) that are protonated in a voltage-dependent manner.

TMEM16A gating in the absence of intracellular Ca^2+^ can be prompted by strong depolarizations, an elevation in temperature, mutating residues Ile637 and Gln645 in the sixth transmembrane segment (Ile641 and Gln649 in our clone) and deleting EAVK segment in the first intracellular loop^23,36–38^. To explain these results, an intrinsic Vm sensitivity in TMEM16A channels has been proposed^37^. However, in TMEM16A an obvious Vm-sensing domain is lacking. Since intracellular acidification reduces TMEM16A activity due to competition between H^+^ and Ca^2+^ for the Ca^2+^-binding pocket^32,33^, we hypothesized that TMEM16A could be gated by Vm-dependent protonation of Glu and Asp residues within the pocket. This is indeed what we found. Our data do not rule out the presence of voltage-sensing domains, instead reveals an unexpected source of Vm dependence, namely Vm dependent protonation. As we acidified the cytosolic side of TMEM16A a large outward I_Cl,Vm_ that lacked time dependence was activated by depolarizations in the absence of intracellular Ca^2+^. The equilibrium constant of protonation and the apparent open probability both increased in a Vm-dependent manner. These effects were reduced after mutating Glu and Asp residues from the Ca^2+^-binding pocket, confirming that H^+^ titrate these residues. Nevertheless, in TMEM16A-5M a channel with all the residues mutated, intracellular acidification enhanced channel activity although the effect was Vm-independent. The estimated *pKa* of this secondary activation is between 4.5 and 6 suggesting protonation of His and probably Asp residues located in the cytosolic side. Together, our data is consistent with a mechanism depicted by the Scheme in Fig. 7.

We propose that intracellular H^+^ ions interact in a Vm-independent manner (*K*_1_ = β_1_/α_1_) with acidic residues (shown in red) located in the cytosolic side of TMEM16A (shown in blue light embedded in a grey membrane; modified from 5OYB^25^) outside the electrical field. A depolarizing stimulus will push H^+^ into the electrical field where they can interact with the acidic residues of the Ca^2+^-binding pocket (*K*_2_ = β_2_/α_2_). Once these residues are protonated (shown in navy), the channels reach the conductive state through a Vm dependent transition (*K*_3_ = β_3_/α_3_) and generate I_Cl,Vm_.

The effects of intracellular H^+^ observed on TMEM16A are comparable to those reported on human SLO1 BK potassium channel^39^. Both channels are activated in the absence of intracellular Ca^2+^ by intracellular acidification and the targets are Ca^2+^ sensing residues. Interestingly, intracellular H^+^ target two His residues, as well as one Asp residue located within the RCK1 domain of BK channels; mutating the His residues, abolished the activating effect of H^+^. In agreement with this, the equilibrium constant of BK protonation has a value of about 6.5 at +100 mV and displays shallow Vm dependence^39^. Here intracellular H^+^ activates TMEM16A by Vm-dependent and independent mechanisms. The Vm-independent *pKa* has a value of about 5.0 at +100 mV, which may suggest titration of Glu, Cys or His residues^40^ just like in BK channels.

Full activation of TMEM16A by intracellular Ca^2+^ is achieved by neutralization of the electrostatic potential generated by the acidic residues of the Ca^2+^-binding pocket and the subsequent movement of TM6 towards TM8^30^. This process seems to be assisted by phosphatidylinositol 4,5 bisphosphate^41–43^. Neutralization of the electrostatic potential by Ca^2+^ is illustrated in TMEM16A Gly644Pro mutant channels where intracellular Ca^2+^ can abolish the strong outward rectification displayed by these channels^30^. In the “constitutively protonated” TMEM16A 5 M channel the electrostatic potential has been neutralized, however, the channel was still activated by Vm and showed outward rectification. This implies that neutralizing the electrostatic potential is not sufficient to abolish rectification. This idea is supported by the strong outward rectification observed under intracellular acidic conditions, which should partially or abolish the electrostatic potential. Alternatively, TM6 may remain bound to TM4 in TMEM16A 5 M thus inducing rectification.

The present work together with a previous report from our group^44^ shows that TMEM16A is the target of extra- and intracellular H^+^. In both cases, protonation of acidic residues enhanced the open probability of the channel albeit extracellular H^+^ do so independently of Vm and Ca^2+^. What would be the physiological consequences of TMEM16A regulation by protons? Although we cannot answer this question yet, we envision that this regulatory process would be important to both TMEM16 channels and scramblases since the residues targeted by H^+^ are present in these proteins. Cancer cells overexpress TMEM16A channels and experience a large pH gradient^20,21,45,46^, conditions that facilitate cell migration and cancer progression. In these cells, the extracellular side is acidic whereas the cytosol is alkaline, and this favours TMEM16A activity. A key salient property of this regulation is that increasing the [H^+^]_e_ or the [H^+^]_i_ increases the current size without changing the fast kinetics. The activation of TMEM16A by Vm in the absence of intracellular Ca^2+^ occurred in less than 1.0 ms, well within the time scale of the electrical activity of excitable cells. Rapid activation of TMEM16A can regulate the electrical activity by inducing membrane depolarization or by accelerating action potential repolarization; this has been shown in neurons from dorsal root ganglia, cholinergic neurons of the medial habenula and muscle cells^36,47^. Thus, activation of TMEM16A enables neurons to respond to thermal stimulus, control anxiety-related behaviour^36,47^ and increase the frequency of action potentials in skeletal muscle cells of zebrafish^48^. A more direct physiological role for TMEM16A regulation by protons is suggested by its simultaneous activation with H^+^ ATPase in the apical membrane of proximal tubules of mouse kidney^49^. In this scenario, a parallel Cl^−^ flux via TMEM16A would serve as a counter ion for H^+^ transport by the V-ATPase. Also, it is interesting to notice that the dimeric channels TMEM16A and CLC-0 are both activated by intracellular to protons^50^. In conclusion, we propose that intracellular H^+^ endow TMEM16A with Vm gating in the absence of intracellular Ca^2+^ by a mechanism that includes Vm-independent titration of cytosolic residues and Vm-dependent titration of acidic residues located in the Ca^2+^-binding pocket.

## Methods

### Cell culture and protein expression

Human embryonic kidney 293 cells (HEK-293) were cultured in Dulbecco’s modified Eagle medium (DMEM, GIBCO BRL) supplemented with 10% FBS and 0.1% penicillin-streptomycin at 37 C° in a 95% O_2_/5% CO_2_ atmosphere. Wild type mouse TMEM16A (ac) and mutant DNAs were cloned into pIRES2-EGFP (Clontech, Mountain View, CA, USA) or pEGFP-N1 vectors. Mutations were introduced using the Quick-change kit (Aligent) and verified by sequencing. HEK-293 cells were transfected with 1 µg/µl cDNA using Polyfect transfection reagent (QIAGEN), according to the manufacturer’s instructions. Cells were used 12 h after transfection. For whole cell recordings, we seeded cells at low density whereas for inside-out recordings stably transfected cells with TMEM16A or transiently transfected mutants were plated onto poly-l-lysine coated coverslips.

### Chloride current recordings by patch clamp

Vm-activated macroscopic chloride (Cl^−^) currents (I_Cl,Vm_) were recorded at room temperature (21–23 °C) from whole cells or inside-out patches expressing wild type (WT) or mutant TMEM16A channels using the patch clamp technique as we previously reported^44,51^. We selected EGFP fluorescent HEK-293 cells using an inverted microscope equipped with UV illumination. Borosilicate patch pipettes were fabricated using P-97 electrode puller (Sutter Instruments CO.). The electrode resistance was 3–5 MΩ for whole cell or 1–2 MΩ for inside-out patches. The stimulation protocol consisted of Vm steps from −100 to +160 mV delivered every 7 s from a holding potential of −30 mV, followed by a repolarization potential to −100 or +100 mV. I_Cl,Vm_ from inside-out patches were recorded using a 635 ms Vm ramp (−100 and +200 mV). Data were acquired using an Axopatch 200B amplifier and the pClamp10 software (Molecular Devices). I_Cl,Vm_ was filtered at 5 kHz and digitized at 10 kHz while the bath was grounded using 3 M KCl agar-bridge connected to an Ag/AgCl reference electrode. Solutions were applied using a home-made gravity perfusion system.

### Solutions to record chloride currents

The extracellular solution contained (in mM): 139 TEA-Cl, 20 HEPES, 0.5 CaCl_2_, and 110 D-mannitol. We adjusted the pH to 7.3 with TEAOH or NaOH. The external solutions were made hypertonic (380–400 mOsm/kg measured by the vapour pressure point method with a VAPRO, Wescor Inc., South Logan, UT, USA) by adding D-mannitol to preclude activation of endogenous volume-sensitive Cl^−^ currents^52^. To determine the anion selectivity sequence, we replaced 100% Cl^−^ with the desired anion. We constructed concentration-response curves for the blockade of I_Cl,Vm_ by tannic and anthracene-9-carboxylic acids by perifusing standard external solution containing increasing concentrations of the inhibitors. To avoid precipitation of tannic acid, we used an extracellular solution containing 139 mM NaCl instead of 139 mM TEA-Cl.

The standard intracellular solution with [Ca^2+^] = 0 and pH = 7.3 contained (in mM): 40 TEA-Cl, 50 HEPES, EGTA-TEA 25.24 and 85 D-mannitol. To obtain [Ca^2+^] = 0.2 µM, we added 5.24 mM CaCl_2_ to the standard intracellular solution and TEA-Cl was reduced to 30 mM. To test the effect of intracellular [H^+^]_i_ on I_Cl,Vm_, the pH of the standard solution was adjusted to 4.0 with 50 mM tartaric acid; to 4.0, 5.0, and 6.0 with 50 mM MES; to 7.3 with 50 mM HEPES; to 8.0 and 9.0 with 50 mM bicine. The pH was adjusted with TEA-OH, the tonicity was 290–300 mosm/kg and the free [Ca^2+^] was calculated using the MAXCHELATOR program (maxchelator.stanford.edu).

All chemicals were purchased from Sigma-Aldrich Co. St. Louis, MO, USA

### Data analysis

Data were analysed and plotted using pClamp 10 (Molecular Devices) and Origin 9 (Origin Lab, Northampton, MA, USA). Red dotted lines in Figures indicate zero current. I_Cl,Vm_ values at each Vm were measured at the end of each depolarization and then corrected for cell size using the cell capacitance. Alternatively, I_Cl,Vm_ values were normalized against the current measured at +160 mV. Normalized I_Cl,Vm_ values were then averaged.

The effect of different blocker concentrations ([B]) or [H^+^]_i_ on I_Cl,Vm_ was quantified from concentration-response curves using the Hill Eq. 1 assuming that *N* is the number of H^+^ or B interacting with one channel according to the following Scheme I:

$$C+N{\rm{B}}\begin{array}{c}\alpha \\ \rightleftharpoons \\ \beta \end{array}C{B}_{N}\,or\,C+N{{\rm{H}}}^{+}\begin{array}{c}k\\ \rightleftharpoons \\ {k}_{-1}\end{array}C{H}_{N}$$

1 $$Response=({I}_{max}-{I}_{min})\frac{1}{1+{\left(\frac{I{C}_{50}}{[B]}\right)}^{N}}+{I}_{min}$$

where I_max_ and I_min_ are the maximum and minimum response, IC_50_ is the concentration of inhibitor ([B]) needed to obtain half I_max_ − I_min_ inhibition; it also represents the equilibrium constant of protonation *K*, and N is the Hill coefficient. For the Vm dependence of K, we fitted titration curves at different Vm, the corresponding K was converted to pK (−log K) and plotted against Vm. The curve was fit with Eq. 2 ^53–55^:

2 $$pK(Vm)=p{K}_{0}+\frac{z\delta FVm}{2.303RT}$$

where *pK*_0_ is the effective pH_i_ needed to obtain half-activation when Vm = 0 mV, R is the gas constant, T is absolute temperature, F is Faraday’s constant, z is the charge, δ is the electrical distance from the inside. To obtain the V_0.5_ value of TMEM16A-WT, the current-voltage curve was adjusted to the following equation:

3 $$y=\frac{(Vm-Vr)\ast {G}_{max}}{1+{e}^{(Vm-{V}_{0.5})/dx}}$$

where Vm is the clamping voltage, Vr is the reversal potential, V_0.5_ is the voltage at which the 50% of channels were activated, G_max_ is the estimated maximum conductance and *dx* is the Vm sensitivity. We estimate the liquid junction potentials using the Clampex routine of pClamp and used them to correct the reversal potential values.

Pooled data are presented as mean ± S.E.M. of n (number of independent experiments). Statistically significant differences between means were determined using a Student t-test or ANOVA.

## Supplementary information

Supplementary Information.
