# Supplementary material for: Voltage-Dependent Protonation of the Calcium Pocket Enable Activation of the Calcium-Activated Chloride Channel Anoctamin-1 (TMEM16A)
Source: Sci Rep. 2020 Apr 20;10:6644. doi: 10.1038/s41598-020-62860-9 (PMC7170896; doi:10.1038/s41598-020-62860-9)
Supplement: Supplementary file 1 — Supplementary Information. [file 41598_2020_62860_MOESM1_ESM.docx]

**VOLTAGE-DEPENDENT PROTONATION OF THE CALCIUM POCKET ENABLE ACTIVATION OF THE CALCIUM-ACTIVATED CHLORIDE CHANNEL ANOCTAMIN-1 (TMEM16A)**

**(SUPPLEMENTARY INFORMATION)**

Guadalupe Segura-Covarrubias^1^, Iván A. Arechiga-Figueroa^3^, José J. De Jesús-Pérez^2^, Alfredo Sánchez-Solano^2^, Patricia Pérez-Cornejo^3^, and Jorge Arreola^2*^

^1^Division de Biología Molecular del Instituto Potosino de Investigación Científica y Tecnológica. Camino a la Presa de San José 2055, San Luis Potosí, SLP 78216, México.

^2^Physics Institute, Universidad Autónoma de San Luis Potosí, Ave. Dr. Manuel Nava #6, San Luis Potosí, SLP 78290 México

^3^Department of Physiology and Biophysics, Universidad Autónoma de San Luis Potosí School of Medicine, Ave. V. Carranza 2405, San Luis Potosí, SLP 78290 México

Corresponding Author: Dr. Jorge Arreola, [arreola@dec1.ifisica.uaslp.mx](mailto:arreola@dec1.ifisica.uaslp.mx)





**Figure S1. Anion selectivity sequence based on the conductivity of WT TMEM16A in the presence of 0.2 µM Ca^2+^.**

A. Representative recordings obtained from cells expressing TMEM16A. The cells were dialyzed with a solution that contained 0.2 μM Ca^2+^ and 40 mM of Cl^-^ and placed in a solution containing 140 mM of the indicated anions.

B. Current - Vm relationships in the presence of Cl^-^ (control) and then switched to a solution with a different anion (test), were obtained from the same cell. We normalized each pair of current- Vm relations to the current magnitude obtained in the presence of Cl^-^ at +160 mV and then averaged the normalized values. (n=6-8). The resulting anion selectivity sequence was determined at +160 mV as the ratio of current in the presence of anion X /current in the presence of Cl^-^. The selectivity sequence was: SCN^-^ (4.24, n=6) >> I^-^ (2.71, n=4) >> NO_3_^-^ (2.70, n=5) >> Br^-^ (1.36, n=5).





**Figure S2. Intracellular acidification does not activate outward rectifying currents in HEK-293 cells.**

A. Representative recordings from untransfected HEK-293 cells dialyzed with 40 mM Cl^-^, 0 Ca_2+_ and 10 mM BAPTA-K (left) or 25.24 mM EGTA-TEA (right) at pH_i_=4.0. Cells were stimulated with the activation protocol shown in Figure 1G. The dotted red lines indicate zero current.

B. Current - Vm curves from experiments like those shown in A using BAPTA-K pH_i_=4 (grey), EGTA-TEA pH_i_=4 (black), EGTA-TEA pH_i_=5 (pink), and EGTA-TEA pH_i_= 7.3 (green) (n=5-6).

C. I_Cl,Vm_ recordings from HEK-293 cells expressing 5M channels. The cells were dialyzed with 40 mM Cl^-^, 10 mM BAPTA-K (left) or 40 mM Cl^-^ and 25.24 mM EGTA-TEA (right) at pH_i_=4. Cells were stimulated with the activation protocol shown in Figure 1G. The dotted red lines indicate zero current.

D. Current - Vm curves from HEK-293 cells transfected with 5M channels. Cells were dialyzed with 40 mM Cl^-^, pH_i_=4, and 0 Ca^2+^ using BAPTA-K (purple, n=5) or EGTA-TEA (black, n=6).





**Figure S3. Voltage activated TMEM16A channels do not display Ohmic behaviour.**

A. Left: Representative recordings of I_Cl,Vm_ from a cell expressing WT TMEM16A channels. The Vm protocol used to activate I_Cl,Vm_ is shown below. Right: Representative I_Cl,Vm_ recordings obtained with the Vm protocols shown below. The cell was dialyzed with [Ca^2+^]_i_ = 0 and [Cl^-^]_i_ 40 mM Cl^-^. The dotted lines represent the zero current. We measured the current at the time indicated by arrows in order to build the current-voltage relationships plotted in B.

B. Instantaneous tail currents – Vm (blue, n= 3) is not linear and the tail current – Vm relationships was Vm-independent (orange, n= 6).





**Figure S4. Vm-dependent protonation of WT TMEM16A channels in two inside-out patches.**

A. Titration curves obtained at voltages from +60 to +200 mV. Curves were constructed using I_Cl,Vm_ recorded from two inside-out patches of obtained from HEK-293 cells expressing WT channels. Continuous lines are fits with the Hill equation.

B. Vm dependence of the equilibrium constant of protonation determined from data shown in A. The continuous lines are the fits with Equation 2 (see main text). The computed δ/K0 values were 0.096 ± 0.005/2.17 ± 0.07 for patch 1 and 0.090 ± 0.007/2.06 ± 0.09 for patch 2.
